# Micromachined Edge Reflection Thin-Film SAW Resonator on LiNbO_3_-on-SiC

**DOI:** 10.3390/mi17091075

**Published:** 2026-09-11

**Authors:** Yu-Hao Wang, Zhen-Hui Qin, Yi-Han He, Hao Yan, Nan-Xin Yu, Cheng-Zhe Cao, Hua-Yang Chen, Si-Yuan Yu, Yan-Feng Chen

**Affiliations:** 1 National Laboratory of Solid State Microstructures, Department of Materials Science and Engineering, Nanjing University, Nanjing 210093, China; 522025340072@smail.nju.edu.cn (Y.-H.W.); zhenhuiqin@smail.nju.edu.cn (Z.-H.Q.); 602023340039@smail.nju.edu.cn (Y.-H.H.); ya_hoo383@163.com (H.Y.); 602024340069@smail.nju.edu.cn (N.-X.Y.); chzhc@smail.nju.edu.cn (C.-Z.C.); 602023340027@smail.nju.edu.cn (H.-Y.C.); 2 Collaborative Innovation Center of Advanced Microstructures, Nanjing University, Nanjing 210093, China; 3 Jiangsu Key Laboratory of Artificial Functional Materials, Nanjing University, Nanjing 210093, China

**Keywords:** edge reflection resonator, LiNbO_3_-on-SiC, miniaturization, SAW resonator, spurious mode suppression, thin-film SAW

## Abstract

This work presents the design and experimental demonstration of a micromachined edge reflection thin-film surface acoustic wave (SAW) resonator on an X-cut LiNbO_3_-on-SiC platform. Unlike conventional Bragg reflection resonators (BRRs), the demonstrated device employs etched free edges adjacent to the interdigital transducer (IDT) to provide lateral acoustic confinement without additional reflector arrays. Edge half electrodes are further used to tailor the lateral phase condition and suppress spurious modes. The representative Design I resonator exhibits an electromechanical coupling coefficient (*k_t_*^2^) of 37.6% and a maximum Bode-Q of 712 at 4.64 GHz, corresponding to a figure of merit (FoM) of 267.7. Compared with BRR counterparts fabricated in the same batch, the ERR reduces the effective resonator footprint by approximately 27–37% while maintaining a comparable *k_t_*^2^. Measurements further show that the ERR exhibits a higher *Q* in most paired designs, thereby yielding an improved FoM. These results establish micromachined edge boundary engineering as an effective route toward reflector-less thin-film SAW resonators with reduced effective resonator footprints and high-Q coupling performance while also clarifying the fabrication-related trade-offs associated with etched free edge confinement.

## 1. Introduction

As wireless communication systems such as 5G-Advanced, 6G, and Wi-Fi 7 continue to evolve toward higher frequencies, wider bandwidths, and higher integration density, increasingly stringent requirements are being imposed on the acoustic resonators used in RF front-end systems [1,2,3]. As a key building block of RF filters, surface acoustic wave (SAW) resonators are widely employed because of their small footprint, low loss, and relatively simple fabrication [4,5]. For next-generation communication applications, SAW resonators are expected to simultaneously provide high operating frequency, a high electromechanical coupling coefficient, a high quality factor, and low spurious response [6,7,8,9]. In recent years, thin-film SAW technologies based on heterogeneous integration platforms have further expanded the design space for meeting these requirements [10,11,12,13]. In particular, high-performance SAW resonators based on LiNbO3-on-SiC have recently been reported, indicating the strong potential of this platform for high-frequency and large-bandwidth RF applications [14,15,16,17,18,19]. LiNbO_3_-on-SiC resonators have also been incorporated into hybrid wideband filters, illustrating the compatibility of this platform with circuit-level bandwidth and selectivity engineering [20]. Nevertheless, under the condition of maintaining these key performance metrics, further reducing resonator size remains highly important for highly integrated RF front-end systems [21].

In conventional SAW resonators, lateral acoustic confinement is typically achieved by placing periodic Bragg reflectors on both sides of the interdigital transducer (IDT) [22]. Although this approach can effectively suppress lateral acoustic leakage, the additional reflector arrays increase the lateral footprint and thus limit further miniaturization. To address this issue, free edge or edge reflection resonators have long been explored as an alternative to conventional reflector-based structures, in which acoustic confinement is achieved through reflection at free edges rather than by Bragg gratings [23]. Kadota et al. employed free edge confinement and half-width terminal electrode fingers to realize compact resonators and filters without grating reflectors on bulk PZT and 36° YX-LiTaO_3_ substrates [24]. More recently, this concept has also been extended to thin-film and micromachined implementations, including resonators employing micromachined or etched vertical boundaries to realize free edge confinement. In addition, Gong and Piazza structurally optimized LiNbO_3_ laterally vibrating MEMS resonators using a weighted electrode configuration, improving the electromechanical coupling coefficient and FoM without relying on external reflector gratings [25]. These studies further demonstrated the feasibility of compact reflector-free SAW resonators and, in some cases, showed improvements in quality factor, transverse mode behavior, and footprint compared with Bragg mirror counterparts [26,27,28]. However, this compactness is obtained by replacing periodic Bragg reflectors with etched free edges, making the device response more sensitive to sidewall angle and the designed relative position between the etched boundary and the edge half electrode. Therefore, its implementation and performance trade-offs in LiNbO_3_-on-SiC thin-film SAW resonators, especially in direct comparison with conventional Bragg reflection resonators (BRRs), remain worthy of further investigation.

In light of these previous studies, the novelty of the present work does not lie in the edge reflection concept or the use of half-width edge electrodes alone. Rather, it lies in their platform-specific co-design and systematic investigation of a high-frequency Love mode ERR based on X-cut LiNbO_3_-on-SiC. First, locally etched LiNbO_3_ free edges that retain the underlying SiC substrate are integrated with symmetric half-width edge electrodes (*W_e_*/2). Comparative simulations are performed to distinguish the roles of the free edges in establishing lateral acoustic confinement from those of the edge electrodes in determining the lateral phase condition. The geometrical parameters of both components are then optimized to enhance acoustic confinement and suppress spurious modes. Second, the fabricated ERRs are directly benchmarked against BRR devices fabricated in the same batch. The comparison involves optimizing the number of BRR reflectors and conducting paired evaluations across multiple IDT periods and propagation directions. The results indicate a 27–37% reduction in effective resonator footprint while maintaining comparable *k_t_*^2^ and yielding a higher maximum Bode-*Q* for most paired designs. Third, finite element simulations are employed to investigate the effects of nonvertical etched sidewalls, while experiments using different designed offsets are conducted to evaluate the impact of the relative displacement between the etched boundary and the edge electrode. These analyses evaluate the influence of two representative fabrication imperfections, namely sidewall geometry and lithographic misalignment, on resonator performance, thereby providing insight into the fabrication tolerance of the proposed structure under practical process variations. Accordingly, the primary contribution of this work is the mechanism-based co-design and integrated numerical–experimental assessment of the trade-offs among footprint, performance, and fabrication tolerance in edge reflection resonators on the LiNbO_3_-on-SiC platform, rather than the introduction of edge reflection or edge electrode engineering as isolated concepts.

## 2. The Design of the Edge Reflection Resonator

To evaluate the performance and compactness of the proposed ERR on the LiNbO_3_-on-SiC platform, this work compares it with a recently reported representative BRR-based SAW resonator as a benchmark. Dai et al. proposed a coupled shear surface acoustic wave (CS-SAW) resonator based on an X-cut LiNbO_3_-on-SiC platform in 2024 [15]. By optimizing the Euler angle and the thickness of the LiNbO_3_ film, the fabricated device achieved a *k*_eff_^2^ of 34% (*k_t_*^2^ of 42.7%) and an FoM of 221 (277.6) near 5 GHz, providing a new perspective for the design of high-performance SAW resonators. However, that work still relied on a Bragg reflector design for lateral acoustic energy confinement and did not further explore device miniaturization from the perspective of edge reflection engineering. Related LiNbO_3_-based heterogeneous SAW studies have addressed Rayleigh, transverse, and higher-order spurious modes through the optimization of multilayered substrates, electrode configurations, and acoustic dispersion [17,18].

**Figure 1:**
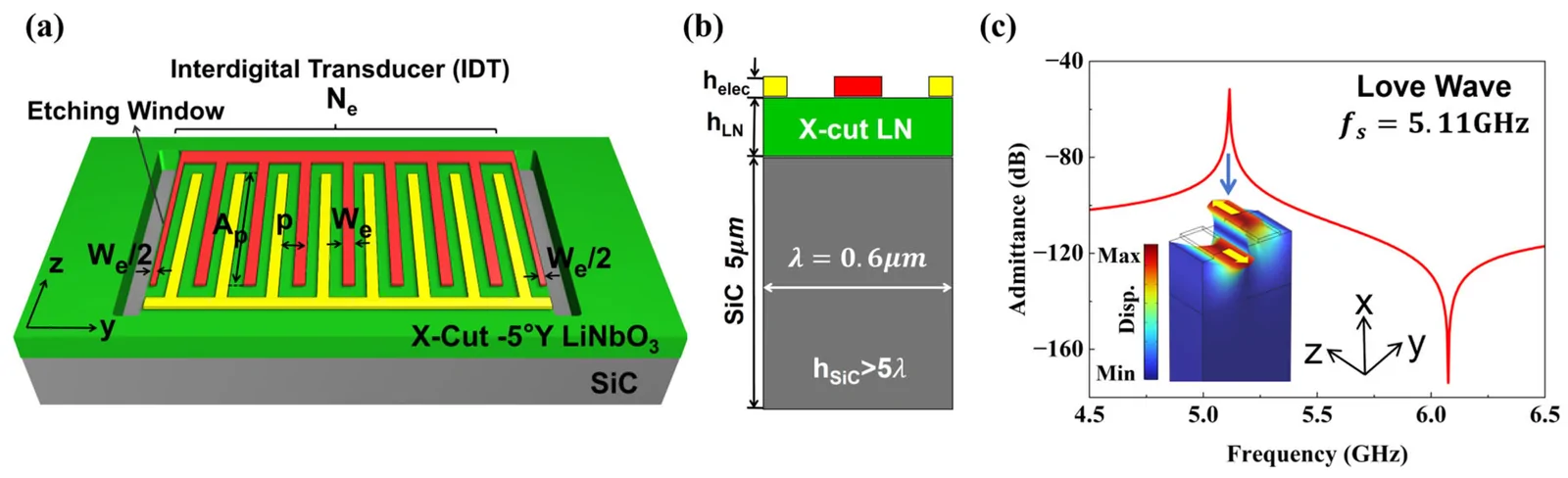
Schematic structure and simulation results of proposed edge reflection resonator (ERR). (**a**) Three-dimensional device schematic with key geometric parameters. (**b**) Unit cell model. (**c**) Simulated admittance spectrum and displacement distribution.

On the same X-cut LiNbO_3_-on-SiC heterogeneous integration platform, this study further introduces an edge reflection design to achieve effective lateral acoustic energy confinement. Figure 1a shows the three-dimensional schematic of the proposed ERR, with its key geometric parameters listed in Table 1. The ERR is configured as a one-port resonator, with a harmonic voltage applied to one IDT busbar and the other busbar grounded. All IDT electrodes connected to the same busbar are treated as equipotential, and the electrical phase delay along the busbar was neglected because the RF guided wavelength is much larger than the aperture length of the present device. All exposed surfaces of the LiNbO_3_ film, including the etched sidewalls, are assigned traction-free mechanical boundary conditions, whereas low-reflecting boundary conditions are applied to the lateral and bottom surfaces of the SiC substrate. The ERR and BRR models use identical material properties, substrate dimensions, excitation conditions, boundary conditions, and meshing criteria, differing only in their lateral acoustic confinement structures. In this structure, the X-cut LiNbO_3_ piezoelectric film is bonded onto a 6H-SiC substrate. The IDT consists of *N_e_* = 20 pairs of metal fingers, with a finger width *W_e_* of λ/4 and an aperture *A_p_* of 20 μm. The acoustic propagation direction is aligned along the −5°Y direction of the X-cut LiNbO_3_ film to enhance the coupling of the fundamental Love mode [29]. Additional orientation sweeps for the ERR and BRR are provided in Figures S1 and S2, respectively, indicating that the change from Bragg reflectors to etched free edges does not shift the optimized −5°Y propagation direction under otherwise identical material and simulation settings. Figure 1b depicts the cross-sectional view of the unit cell model of the ERR. The wavelength λ is set to 0.6 μm, the thickness of the LN layer (*h*_LN_) is set to λ/2, and Au (20 nm) is selected as the electrode material [30]. Based on finite element method (FEM) simulations and optimization, the simulated admittance response and displacement distribution of the ERR are as shown in Figure 1c. The simulated displacement distribution confirms the excitation of the target fundamental Love mode.

Unlike conventional Bragg-reflector-based schemes, the proposed approach introduces the edge reflection free boundary design. Specifically, while preserving the integrity of the SiC substrate, the LiNbO_3_ layer on both sides of the IDT is locally etched to form free boundaries, thereby enhancing the lateral acoustic impedance mismatch. As a result, strong acoustic reflection can still be generated at the boundary without requiring additional reflector regions, which enables device miniaturization. Moreover, the effective reflection of a Bragg reflector grating relies on the finite stopband formed by its periodic structure, whereas a free boundary does not require Bragg phase matching and can provide acoustic wave reflection over a broader frequency range. Based on this edge reflection structure, two symmetric edge half electrodes, each with a width of *W_e_*/2, are further introduced at both edges of the IDT. This can be understood as forming a standing wave structure analogous to a mechanical Fabry–Perot (FP) cavity, which modulates the electromechanical boundary conditions at the lateral edges, thereby optimizing the phase matching condition of the fundamental mode and suppressing spurious modes [31,32,33]. To systematically evaluate the feasibility of the proposed design, FEM simulations in COMSOL Multiphysics 6.3 were performed to compare and analyze the following four configurations: (1) a conventional BRR with Bragg reflectors; (2) the proposed ERR with edge reflection and an equal width half electrode (*W_e_*/2); (3) edge reflection with an equal width edge electrode, where *d*_1_ = *d*_2_ ≠ *W_e_*/2; and (4) edge reflection with an unequal width edge electrode, where *d*_1_ + *d*_2_ = *W_e_*.

To quantitatively compare the spurious mode responses, the absolute spurious mode metric *M* proposed by Colombo et al. [34] was calculated as

(1) $$M=\sum_{j=1}^{n}\left|maxY_{j}-minY_{j}\right|$$

where *n* is the number of spurious modes, and max(*Y_j_*) and min(*Y_j_*) are the admittance levels at the resonance and anti-resonance of the *j*-th spurious mode, respectively. In this work, only parasitic responses exhibiting a resonance-to-anti-resonance admittance difference greater than 1 dB were identified as spurious modes and included in the calculation of *M*. Therefore, a lower *M* value indicates weaker overall spurious mode responses and, consequently, better spurious mode suppression.

**Figure 2:**
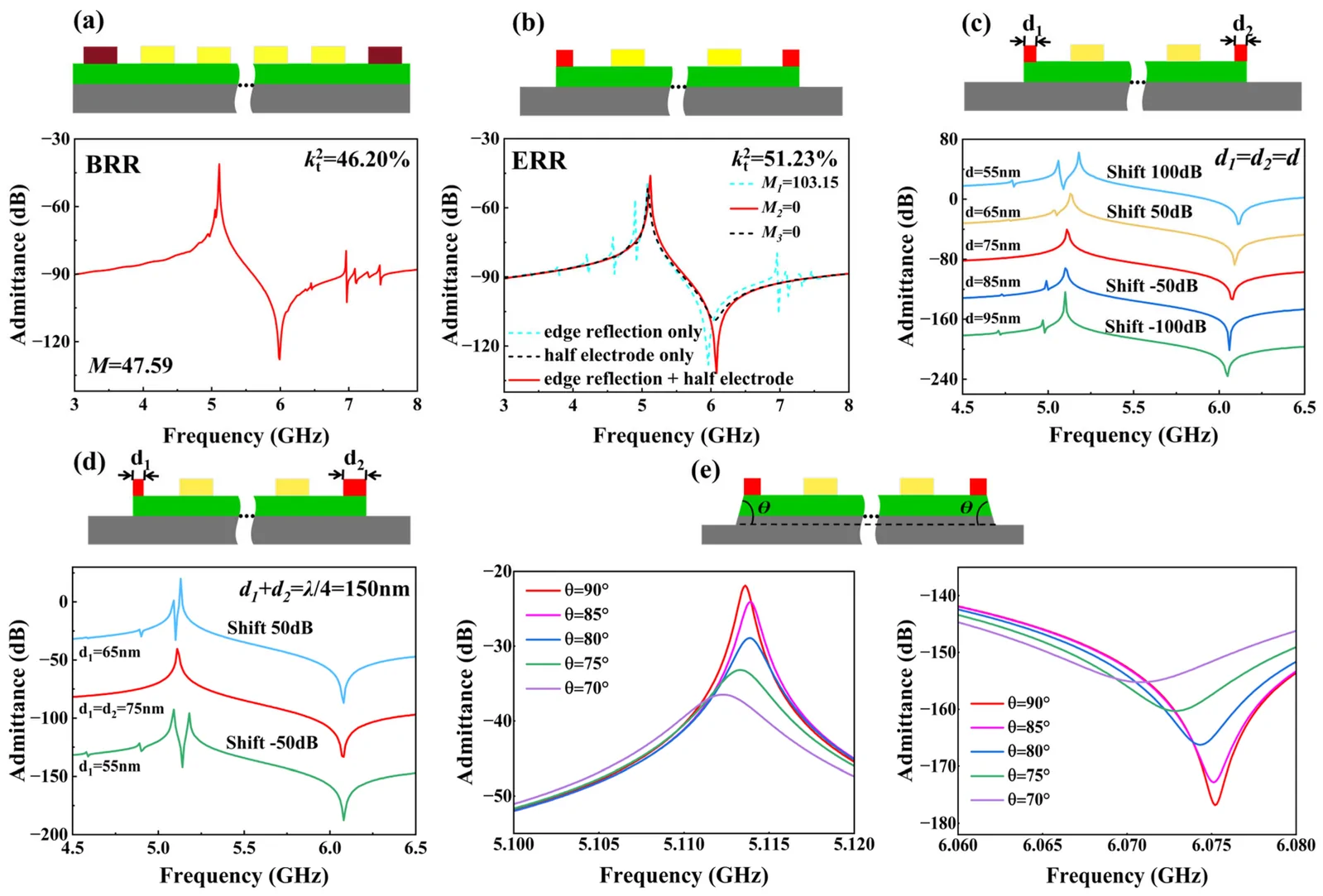
Analysis and simulation of lateral acoustic confinement in edge reflection resonator (ERR). (**a**) Bragg reflection resonator (BRR) structure. (**b**) ERR structure and comparison of admittance responses for edge-reflection-only design, edge-half-electrode-only design, and their combined design. (**c**) Effect of different edge electrode widths on admittance responses when *d*_1_ = *d*_2_. (**d**) Effect of asymmetric edge electrode dimensions on admittance responses when *d*_1_ + *d*_2_ = *W_e_*. (**e**) Effect of etch sidewall angle on admittance responses.

Figure 2a,b show the simulated admittance responses of the BRR and ERR devices under the same design parameters. In the figures, brown, yellow, and red denote the Bragg reflectors, the IDT electrodes, and the edge electrode. The simulated wideband responses in Figure 2a,b show that the BRR exhibits several additional out-of-band responses, whereas the ERR configuration maintains a smoother response outside the main resonance and anti-resonance. Compared with the BRR, the ERR exhibits a cleaner admittance response, with *M* decreasing from 47.59 dB to 0 dB. This zero value indicates that no spurious modes in the ERR exceed the 1 dB criterion. This improvement mainly arises from the free boundary introduced by the edge reflection structure and the modulation of the lateral phase condition by the half electrode structure, which together lead to the stronger localization of acoustic energy within the active IDT region. In addition, removing the Bragg reflector electrodes eliminates reflector-associated mechanical and electrical perturbations outside the active IDT region. Related electrode engineering approaches have also been reported for suppressing transverse spurious modes in LiNbO_3_ acoustic resonators [35]. To further clarify the individual roles of the edge reflection and the half electrode, Figure 2b also includes the auxiliary responses of the edge-reflection-only design (blue dashed line) and the half-electrode-only design (black dashed line). The results show that the edge-reflection-only design already provides strong lateral confinement, although obvious undesired spurious modes still remain near the main resonance peak. In contrast, for the resonator employing only the half electrode design, the spurious modes are significantly suppressed [36]. However, the admittance response in the vicinity of the anti-resonance becomes significantly flattened, indicating that the half electrode alone is insufficient to provide adequate lateral acoustic energy confinement. This work therefore adopts a design strategy that combines edge half electrodes with the edge reflection structure, thereby realizing a high-performance SAW resonator without pronounced spurious modes.

To further investigate the influence of edge electrode geometry on spurious modes, Figure 2c,d present the admittance responses for different electrode dimensions. Specifically, Figure 2c examines symmetric edge electrode width variations with *d*_1_ = *d*_2_, whereas Figure 2d examines asymmetric width distributions under a fixed total width of *d*_1_ + *d*_2_ = *W_e_*. The comparison results show that when the edge electrode width satisfies the ideal symmetric condition *d*_1_ = *d*_2_ = *W_e_*/2 (the red line), the device exhibits the least perturbed main resonance response and achieves the best suppression of spurious modes. In contrast, when the edge electrode dimensions deviate from the ideal symmetric condition, the suppression of spurious modes is degraded. The physical function of the edge half electrodes can be attributed to boundary phase matching between the electrical excitation profile and the laterally reflected acoustic field. Since the IDT finger width is *W_e_* = *λ*/4, the symmetric condition *d*_1_ = *d*_2_ = *W_e_*/2 forms half-width terminal electrodes at both etched free edges. This configuration provides symmetric electrical termination at the two acoustic reflection boundaries, which favors the lateral standing wave condition of the target Love mode and reduces the excitation of mismatched transverse modes. When the edge electrodes are removed, widened, narrowed, or made asymmetric, this boundary phase matching is degraded, resulting in stronger spurious modes. Symmetric edge half electrodes satisfying *d*_1_ = *d*_2_ = *W_e_*/2 are therefore more favorable for strengthening the fundamental mode and suppressing mismatched spurious modes [37].

In addition to the geometric design of the edge electrode, the non-ideal sidewall etching of the LiNbO_3_ thin film during practical fabrication can also significantly affect device performance [38]. To investigate this factor, simulations were further conducted for different etch sidewall angles, and the corresponding variation in admittance response is shown in Figure 2e. The results indicate that as the etch sidewall angle gradually approaches 90°, the resonance and anti-resonance peaks become progressively sharper, with a corresponding increase in quality factor. This behavior arises because non-ideal boundaries modify the boundary conditions, causing part of the acoustic wave to undergo scattering or mode conversion, thereby reducing the boundary reflection efficiency and leading to energy leakage into the substrate or surrounding regions. Therefore, improving sidewall verticality is of great importance for achieving favorable lateral reflection conditions and realizing high-*Q* devices.

## 3. Measurement Results

**Figure 3:**
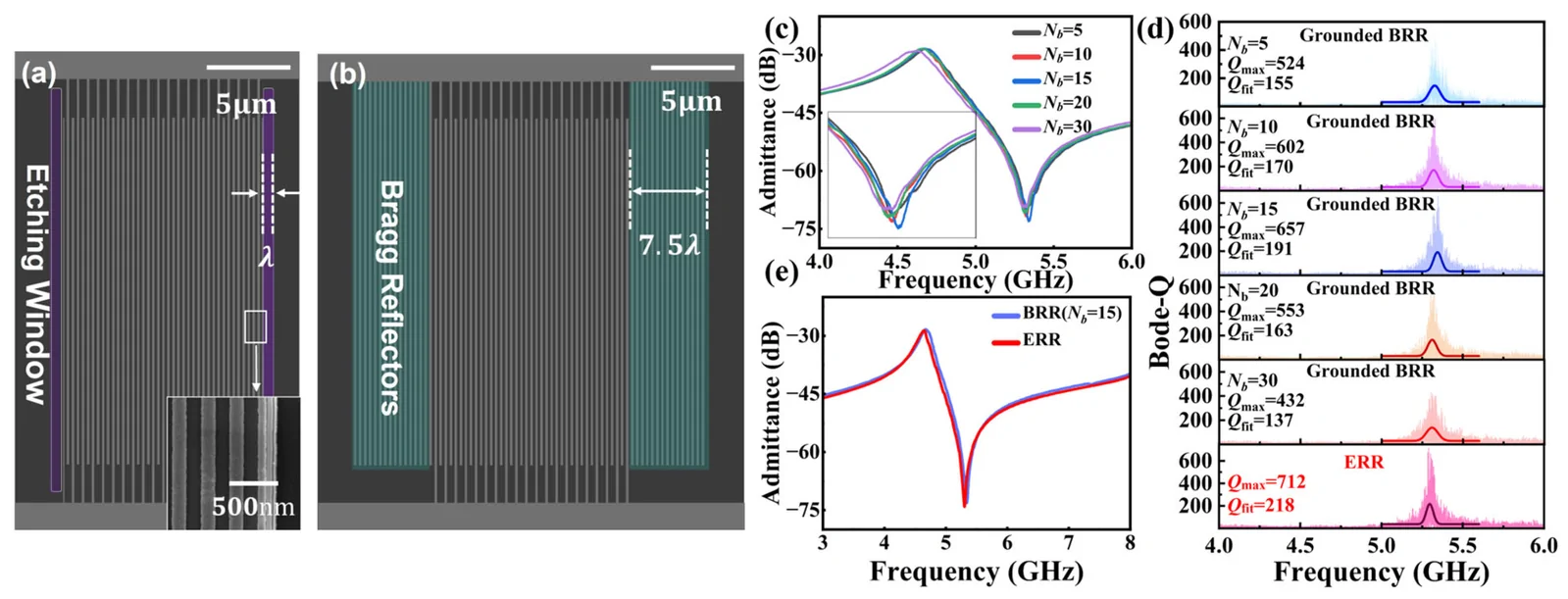
(**a**,**b**) show SEM images of fabricated ERR and BRR, respectively. (**c**) Measured admittance responses of BRR devices with different numbers of reflector pairs. (**d**) Bode-Q responses and Gaussian fits of BRR devices with different reflector pair numbers and ERR device. (**e**) Comparison of admittance responses of BRR with *N_b_* = 15 and ERR.

To verify the practical feasibility of the ERR design and its performance advantages over the BRR, an edge reflection Love mode resonator based on a 300 nm X-cut LiNbO_3_-on-SiC structure was fabricated, and its scanning electron microscope (SEM) image is shown in Figure 3a. The SEM image shows the fabricated ERR structure. The IDT consists of 20 pairs of metal fingers, with a period of 0.6 μm and an aperture of 20 μm. Equal width edge half electrodes with a width of 75 nm are introduced on both sides of the IDT, and the LiNbO_3_ thin film is locally etched to form a free boundary. A magnified view of the edge reflection region is provided in the lower right corner, where the etched sidewall of the LiNbO_3_ exhibits a certain angle, while the edge reflection boundary remains clearly distinguishable. The etched window adjacent to the edge reflection boundary is approximately one acoustic wavelength wide. The detailed fabrication process flow is provided in Figure S3 in the Supplementary Material.

To ensure a fair performance comparison, grounded BRR devices were also experimentally fabricated under the same conditions, with multiple groups having different numbers of reflector pairs, as shown in Figure 3b [39]. Periodic metal fingers were introduced on both sides of the IDT, with the same period as the IDT to satisfy the Bragg reflection condition. All ERR and BRR devices in Designs I–V were fabricated in the same process batch. Within each paired design, the material stack and IDT parameters were matched, with the lateral acoustic confinement structure being the primary difference. The resonators were measured at room temperature using a vector network analyzer (Ceyear 3671e), and standard short-open-load-through (SOLT) calibration was performed prior to measurement.

The electromechanical coupling coefficient *k_t_*^2^ is defined using the measured resonance frequency *f_s_* and anti-resonance frequency *f_p_* as follows [40]:

(2) $$k_{t}^{2}\;=\;\left(\pi^{2}/8\right)\;\times \;(f_{p}^{2}\;-\;f_{s}^{2})\;/\;f_{s}^{2}$$

The quality factors *Q* are calculated with Bode’s method using the following equation:

(3) $$BodeQ\;=\left[\omega \left|S_{11}\right|delay\left(S_{11}\right)\right]\;/\left(1-{{|S}_{11}|}^{2}\right)$$

where |*S*_11_| denotes the magnitude of the reflection coefficient at port 1, and delay(*S*_11_) denotes the group delay of *S*_11_ [41]. 

The measured responses were calibrated and de-embedded before data analysis. No additional numerical smoothing was applied to the directly calculated Bode-Q curves. To provide an auxiliary assessment of the overall Bode-Q peak trend, the Bode-Q response within the target resonance region was fitted using a single-peak Gaussian function. The maximum value of the fitted curve was defined as *Q*_fit_. The same Gaussian fitting method and a consistent frequency window selection criterion were applied to all ERR and BRR devices. *Q*_fit_ is used only as an empirical indicator of the overall peak trend and does not replace the *Q*_max_ directly extracted from the measured Bode-Q response.

Figure 3c shows the influence of different numbers of reflector pairs (*N_b_* = 5–30) on resonator performance under the same design parameters, with an enlarged view of the anti-resonance peak provided in the lower left corner. The curves in Figure 3d present the measured Bode-*Q* responses and the corresponding single-peak Gaussian fits of the ERR and BRR devices. For each device, the maximum value directly extracted from the measured Bode-*Q* response is denoted as *Q*_max_, whereas the peak value of the Gaussian-fitted curve is denoted as *Q*_fit_. Here, *Q*_fit_ is used only as an empirical auxiliary indicator of the overall Bode-*Q* peak trend and does not replace the directly extracted *Q*_max_. It can be observed that, with increasing reflector pair number, both the *Q*_max_ and *Q*_fit_ of the BRR first increase, then tend to saturate, and finally decrease. When *N_b_* =15, *Q*_max_ = 657 and *Q*_fit_ =191 are obtained. This is because, when the number of reflector pairs is relatively small, increasing *N_b_* helps enhance the reflection strength and thus improves lateral acoustic energy confinement. Beyond the optimum value, however, further increasing *N_b_* no longer improves confinement efficiency and may introduce additional acoustic loss and boundary perturbation, resulting in a reduction in Q. Therefore, the BRR device with *N_b_* =15 was selected for the subsequent comparison, based on the directly extracted *Q*_max_, while *Q*_fit_ is shown only as an auxiliary visualization of the overall peak trend.

Figure 3e compares the admittance responses of the optimized BRR (*N_b_* = 15) and the proposed ERR under the same design parameters. It can be seen that the ERR achieves *Q*_max_ = 712, *k_t_*^2^ = 37.6%, and FoM = 267.7. The experimentally extracted *k_t_*^2^ of 37.6% is lower than that predicted by the ideal FEM model. This difference may partly arise from the nonvertical etched sidewalls (approximately 75°) and parasitic effects associated with the pads and interconnects, which are not fully represented in the idealized model. The simulated influence of the IDT sidewall angle is provided in Figure S4 in the Supplementary Material. Thus, the ideal FEM value should be regarded as an upper-bound estimate for the nominal geometry rather than a direct prediction of the fabricated device. Compared with the BRR (*N_b_* = 15), *Q*_max_ and FoM are improved by 8.4% and 5.6%, respectively, without sacrificing *k_t_*^2^ or operating frequency and without showing pronounced additional spurious responses in the compared admittance spectra. These results show the measured footprint and performance differences between the edge reflection and optimized Bragg reflection implementations. Meanwhile, by comparing Figure 3a,b, it can be seen that because the ERR does not require an additional Bragg reflector region, its effective resonator footprint is reduced by approximately 37% relative to that of the conventional BRR, further demonstrating the potential advantage of the proposed design for device miniaturization. Table 2 summarizes the performance of conventional BRRs with different numbers of reflector pairs, together with that of the ERR. The comparison further suggests that the ERR provides more effective lateral acoustic confinement than the BRR.

**Figure 4:**
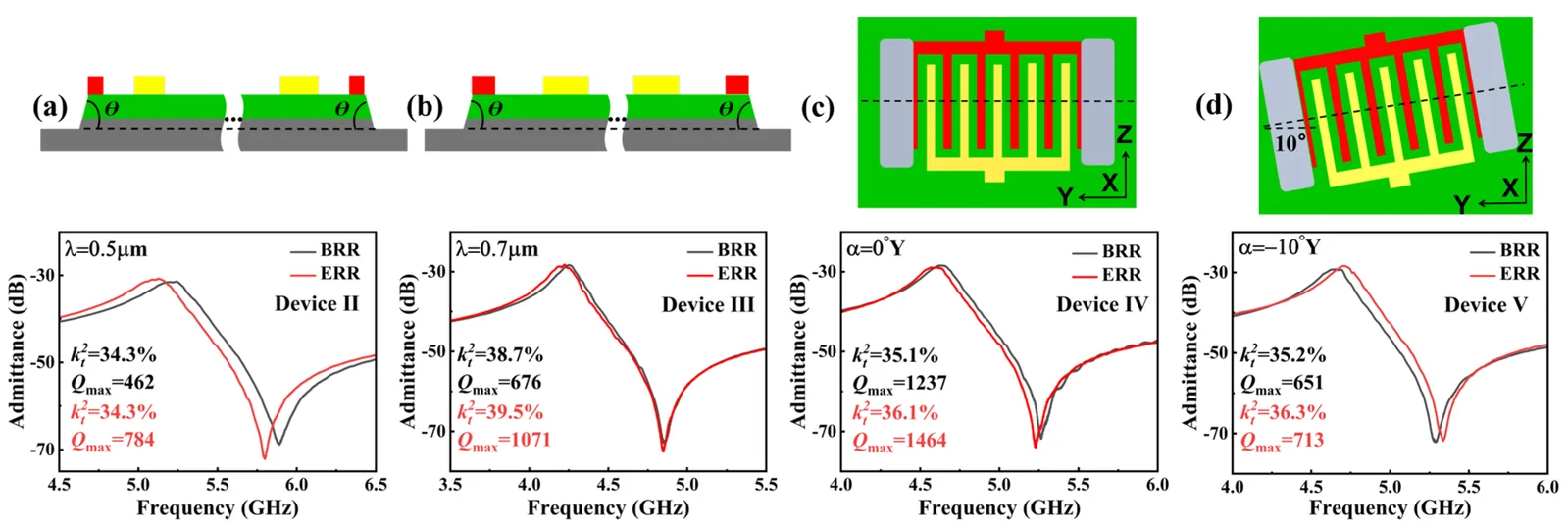
(**a**,**b**) show the schematic structures and measured admittance responses of the devices with IDT periods of 0.5 μm and 0.7 μm along the −5°Y propagation direction. (**c**,**d**) show the schematic structures and measured admittance responses of the devices with a period of 0.6 μm along the 0°Y and −10°Y propagation directions.

To further verify the generality of the ERR structure, additional comparisons between the ERR and BRR were carried out under different periods and propagation directions, and the measured admittance responses are shown in Figure 4. First, Figure 4a,b present the measured results for Design II and Design III, corresponding to devices with a propagation direction of −5°Y and IDT periods of 0.5 μm and 0.7 μm, respectively. It can be observed that, for Design II, the ERR outperforms the BRR: while maintaining nearly the same *k_t_*^2^, *Q*_max_ increases from 462 to 784, corresponding to a 69.7% increase. For Design III, the Love mode can still be effectively excited, and *k_t_*^2^ increases from 38.7% to 39.5%. In addition, *Q*_max_ increases from 676 to 1071, corresponding to a 58.4% increase. 

Figure 4c,d show the measured admittance responses of the devices with a period of 0.6 μm in the 0°Y and −10°Y propagation directions. It can be seen that the ERR structure remains effective under different propagation directions, and both *k_t_*^2^ and *Q*_max_ are improved to a certain extent compared with those of the BRR structure. In Design IV (0°Y propagation direction), the altered coupling among the lateral boundary modes gives rise to several small spurious mode responses in the high-frequency region of the BRR spectrum, whereas the ERR maintains a cleaner admittance response, indicating weaker observed spurious responses for the ERR in this design.

**Figure 5:**
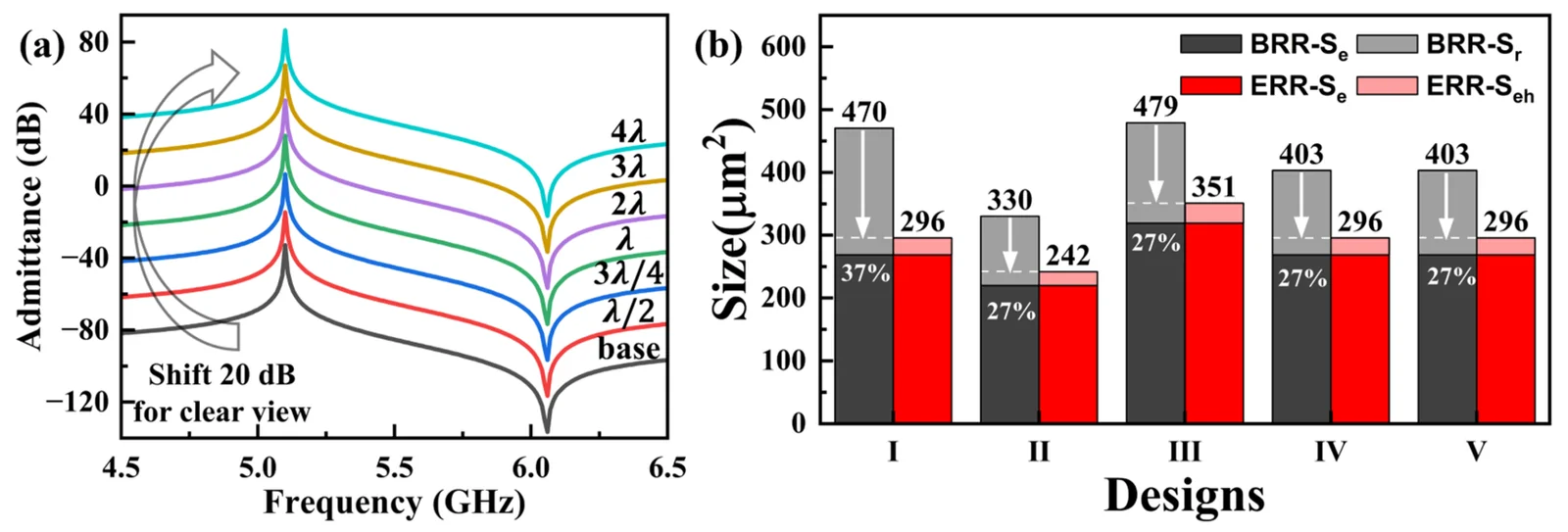
Influence of etching window width and footprint comparison between ERR and BRR designs. (**a**) Simulated admittance spectra of ERR with different etching window widths. Here, “base” represents idealized reference case with effectively infinite etching window width. (**b**) Effective resonator footprint comparison between ERR and BRR for Designs I–V. *S_e_* denotes IDT footprint for both ERR and BRR, *S_r_* denotes Bragg reflector footprint in BRR, and *S_eh_* denotes etched window footprint adjacent to edge reflection boundaries in ERR.

Across the investigated designs, the ERR maintains comparable broadband admittance behavior and shows weaker observed spurious responses in selected configurations while achieving a higher *Q*_max_ in most paired comparisons. To support a fair effective resonator footprint comparison, Figure 5a first examines the effect of the etching window width on the simulated ERR response. The simulated admittance spectra for different etching window widths exhibit nearly identical resonance and anti-resonance frequencies, while the overall spectral line shapes remain largely unchanged. These results indicate that the etching window width has a limited influence on the main resonance characteristics. This trend is consistent with the intended function of the etching window, which primarily provides the process clearance required to define the free edge rather than serving as a dominant acoustic confinement region.

Accordingly, the etching window width can be selected according to the available process margin, and a one-acoustic-wavelength etching window is adopted as the ERR footprint benchmark in Figure 5b. For the effective resonator footprint comparison, the BRR area is defined as the IDT region integrated with the Bragg reflector regions on both sides, whereas the ERR area is defined as the IDT region integrated with the etching windows adjacent to the edge reflection boundaries. Based on this definition, the ERR reduces the effective resonator footprint by approximately 27–37% compared with the corresponding BRR designs while maintaining the performance advantages discussed above. These results demonstrate the miniaturization benefit of the free boundary/edge half electrode ERR design on the LiNbO_3_-on-SiC platform.

The FEM simulations in Section 2 are based on an ideal ERR structure with vertical sidewalls and a zero designed offset (m = 0) between the edge reflection boundary and the edge electrode. However, fabricated devices may deviate from this ideal structure because of process non-idealities, including nonvertical etched sidewalls, fabrication-induced overlay errors between the edge reflection boundary and the edge electrode, and electrode width variation.

**Figure 6:**
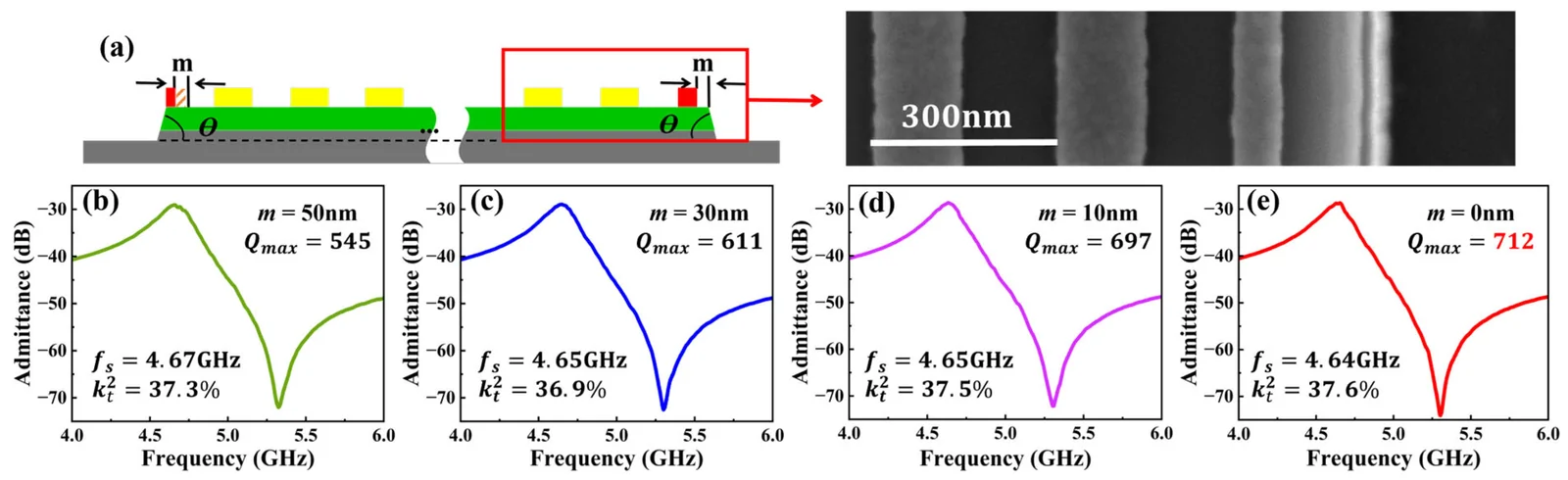
Effect of layout-designed offset *m* on measured response of ERR device. (**a**) SEM image and geometric schematic of ERR boundary region. (**b**–**e**) Measured admittance responses for values of *m*.

Figure 6a shows the geometric schematic of the ERR device together with the corresponding SEM image of the boundary region. The fabricated resonator boundary does not form an ideal vertical sidewall; the measured etched sidewall angle is approximately 75°. Accordingly, in Figure 6, the layout-defined offset m is intentionally varied to examine the sensitivity of the ERR response to the relative position between the edge reflection boundary and the edge electrode under this measured nonvertical sidewall condition.

It should be noted that the layout-defined offset *m* in Figure 6 is a designed offset intentionally introduced at the layout design stage between the edge reflection boundary and the edge electrode, rather than a criterion used to select or group devices according to the actual overlay error after fabrication. Devices with different designed *m* values were fabricated while retaining the nonvertical sidewall angle. Figure 6b–e show the measured admittance responses as *m* decreases from 50 nm to 0 nm. The results show that as *m* decreases, the resonance and anti-resonance peaks become sharper, and *Q*_max_ increases from 545 to 712. This indicates that the lateral acoustic energy confinement is improved as the relative position between the edge reflection boundary and the edge electrode approaches the ideal design. Meanwhile, the extracted *k_t_*^2^ decreases only slightly as *m* increases, indicating that the layout-defined boundary electrode offset has a limited influence on *k_t_*^2^. Even with an approximately 75° nonvertical sidewall, the ERR designed with *m* = 10 nm still achieves *Q*_max_ = 697, which is higher than the *Q*_max_ = 657 of the BRR device. These results indicate that accurate boundary electrode alignment helps improve lateral acoustic energy confinement. The measured trend with the layout-defined *m* is also consistent with the analysis in Figure 2c–e regarding the effects of edge electrode geometry and non-ideal boundary conditions.

Designs I–V represent different device configurations with distinct IDT periods and/or propagation directions and should therefore not be interpreted as replicate samples for device-level statistical analysis. The reported devices were measured repeatedly under the same calibrated measurement conditions, and the resulting admittance spectra showed a high degree of overlap, indicating good measurement consistency.

Finally, Table 3 compares this work with representative TF-SAW resonators near 5 GHz. The results show that the proposed ERR exhibits a relatively high electromechanical coupling coefficient and competitive overall performance among reported devices. In particular, on the same LiNbO_3_-on-SiC platform, the ERR provides a reduced effective resonator footprint compared with reflector-based counterparts without sacrificing performance. This highlights the potential of the edge reflection design for the miniaturized implementation of high-performance SAW resonators. To ensure consistency in the literature comparison, all effective electromechanical coupling coefficients (*k_t_*^2^) in the table were calculated consistently using Equation (2), while all Q values are based on the Bode-Q definition given in Equation (3).

## 4. Conclusions

This work experimentally evaluates a micromachined edge reflection thin-film SAW resonator (ERR). Unlike conventional Bragg reflection resonator (BRR) designs, the ERR achieves lateral acoustic confinement by introducing etched free edges along the lateral direction of the device. Edge half electrodes are further employed to tailor the lateral phase condition and suppress spurious modes while preserving the key performance metrics of the SAW resonator. The structure is investigated through both simulation and experimental validation on an advanced LiNbO_3_-on-SiC thin-film SAW platform. Compared with optimized BRR counterparts, the proposed resonator reduces the effective resonator footprint by approximately 27–37% while maintaining a comparable electromechanical coupling coefficient, and it exhibits a higher *Q* in most paired designs, thereby yielding an improved figure of merit (FoM). In addition, the relationship revealed in this work between non-ideal boundaries and the maximum quality factor provides useful guidance for the structural design and process optimization of edge reflection resonators.

## Figures and Tables

**Table 1:** Key geometric and crystallographic parameters used in FEM model.

Parameter	Value	Parameter	Value
Wavelength (λ)	0.6 μm	Electrode Thickness (*t*_Au_)	20 nm
No. of IDT Pairs (*N_e_*)	20	LN Thickness (*h*_LN_)	300 nm
Aperture (*A_p_*)	20 μm	Euler Angles	(5°, 90°, 90°)
Electrode Width (*W_e_*)	150 nm	Substrate	5 μm SiC
Edge Electrode Width (*W*_edge_)	75 nm	No. of BRR Reflectors (*N_b_*)	15

**Table 2:** Performance comparison between the BRR and ERR devices.

Design I		*f_s_* (GHz)	*k*_t_^2^ (%)	*Q*_max_	FoM
BRR	*N_b_* = 5	4.68	36.6	524	191.8
	*N_b_* = 10	4.66	37.4	602	225.1
	*N_b_* = 15	4.66	38.6	657	253.6
	*N_b_* = 20	4.65	38.1	553	210.7
	*N_b_* = 30	4.62	39.6	432	171.1
ERR	—	4.64	37.6	712	**267.7**

**Table 3:** Comparison of representative TF-SAW resonators.

Reference	Substrate	Mode	*f_s_* (GHz)	*k_t_*^2^ (%)	Bode-*Q*	FoM
[42]	42°YX LT/SiC	LL	4.31	4.6	1180	54.3
[43]	LN/Quartz	LL	6.03	13.6	339	46.1
[11]	128°Y-LN/SiO_2_/p-Si/Si	SV	7.07	8.9	316	28.1
[15]	X-LN/SiC	Love	4.88	42.7	650	277.6
[44]	X-LN/SiC	SH	3.58	32.2	518	166.8
[16]	X-LN/SiC	LL	4.95	20.7	408	84.5
This work (Design I)	X−5°Y LN/SiC	Love	4.64	37.6	712	267.7

All *k_t_*^2^ values were calculated using Equation (2). The Bode-Q values were taken as reported in the cited references; Refs. [11,15,42], together with this work, report Bode-Q values calculated directly from measured responses, whereas Refs. [16,43,44] report values calculated from mBVD-fitted responses.

## Data Availability

The original contributions presented in this study are included in the article and Supplementary Material. Further inquiries can be directed to the corresponding authors.

## Supplementary Materials

The following supporting information can be downloaded at: https://www.mdpi.com/article/10.3390/mi17091075/s1, Figure S1: Simulated admittance responses of the ERR in different propagation directions: (**a**) −3°Y, (**b**) −5°Y, (**c**) −7°Y, (**d**) −10°Y, (**e**) 0°Y, and (**f**) +5°Y. (**g**) Displacement field of the Rayleigh parasitic mode marked by the red box. The target Love-mode response is cleanest along the −5°Y direction; Figure S2: Simulated admittance responses of the BRR in different propagation directions: (**a**) +5°Y, (**b**) 0°Y, (**c**) −5°Y, and (**d**) −10°Y. The BRR also obtains the cleanest target Love-mode response along the −5°Y direction; Figure S3: Schematic fabrication process of the ERR, including composite hard-mask formation, pattern transfer, LiNbO3 etching, mask removal, and IDT electrode fabrication; Figure S4: Simulated admittance responses of the resonator with different IDT sidewall angles. (**a**–**c**) Admittance responses for IDT sidewall angles of 80°, 85°, and 90°, respectively. (**d**) Enlarged view around the antiresonance frequency. The main resonance response remains nearly unchanged, while a slight shift in the antiresonance frequency is observed as the IDT sidewall angle varies.
