# Supplementary material for: Micromachined Edge Reflection Thin-Film SAW Resonator on LiNbO3-on-SiC
Source: Micromachines (Basel). 2026 Sep 11;17(9):1075. doi: 10.3390/mi17091075 (PMC13609639; doi:10.3390/mi17091075)
Supplement: Supplementary file 1 [file micromachines-17-01075-s001.zip › micromachines-4539727-supplementary.pdf]

Micromachined Edge-Reflection Thin-Film SAW Resonator on $LiNbO_3$-on-SiC

Yu-Hao Wang [1,†], Zhen-Hui Qin [1,†], Yi-Han He [1], Hao Yan [1], Nan-Xin Yu [1], Cheng-Zhe Cao [1], Hua-Yang Chen [1], Si-Yuan Yu [1,2,3]* and Yan-Feng Chen [1,2,3]*

1 National Laboratory of Solid State Microstructures, Department of Materials Science and Engineering, Nanjing University, Nanjing 210093, China
2 Collaborative Innovation Center of Advanced Microstructures, Nanjing University, Nanjing 210093, China
3 Jiangsu Key Laboratory of Artificial Functional Materials, Nanjing University, Nanjing, 210093, China
*Correspondence: yusiyuan@nju.edu.cn (S.-Y.Y.); yfchen@nju.edu.cn (Y.-F.C.)
† These authors contributed equally to this work.

This Supplementary Material includes the following parts:

I. Propagation-Direction Optimization for ERR and BRR

II. Fabrication Process of the ERR

III. Influence of IDT Sidewall Angle on Resonator Performance

I. Propagation-Direction Optimization for ERR and BRR

The in-plane propagation direction was swept to identify the orientation that supports the cleanest target Love-mode response for both the edge-reflection resonator (ERR) and the Bragg-reflection resonator (BRR). In these simulations, the material stack, electrode geometry, excitation condition, substrate dimensions, boundary conditions, and meshing criteria were kept unchanged, while only the propagation direction of the X-cut $LiNbO_3$ film was varied. This comparison separates the effect of crystal orientation from the effect of the lateral acoustic confinement structure.

For the ERR, the simulated admittance responses indicate that the -5°Y propagation direction gives the cleanest target Love-mode response among the investigated orientations. Other propagation directions introduce stronger parasitic responses, including the Rayleigh-type parasitic mode marked in Figure S1g. The BRR exhibits the same orientation preference under the corresponding simulation conditions. The -5°Y direction therefore represents the optimized propagation direction for the Love-mode resonator in both the ERR and BRR configurations, indicating that replacing Bragg reflectors with etched free edges does not shift the preferred $LiNbO_3$ propagation direction.

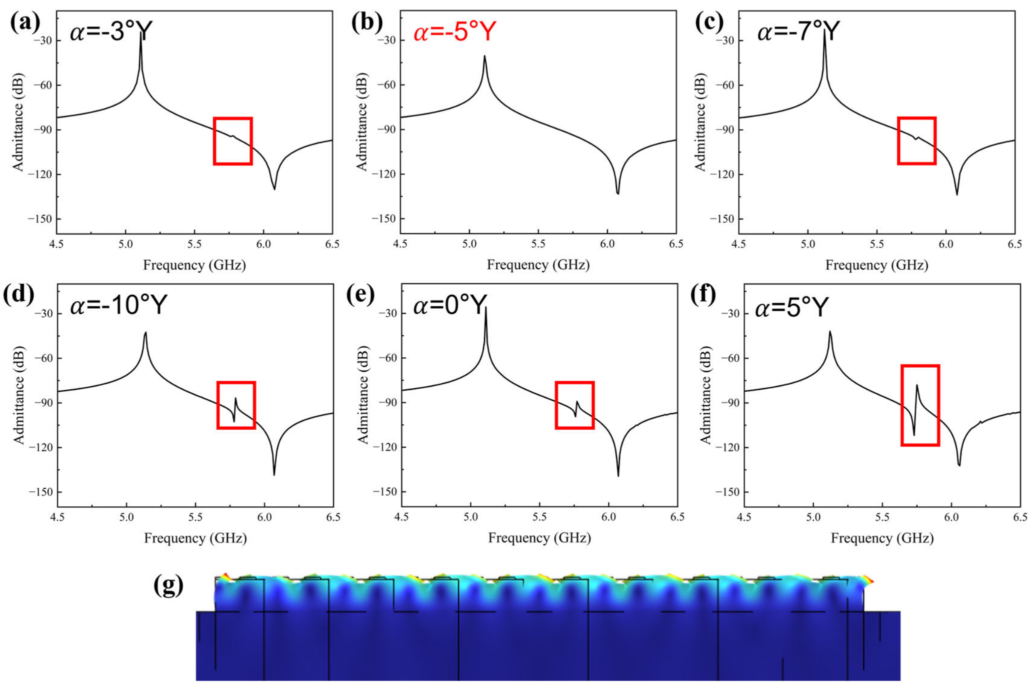

Figure S1. Simulated admittance responses of the ERR in different propagation directions: (a) -3°Y, (b) -5°Y, (c) -7°Y, (d) -10°Y, (e) 0°Y, and (f) +5°Y. (g) Displacement field of the Rayleigh parasitic mode marked by the red box. The target Love-mode response is cleanest along the -5°Y direction.

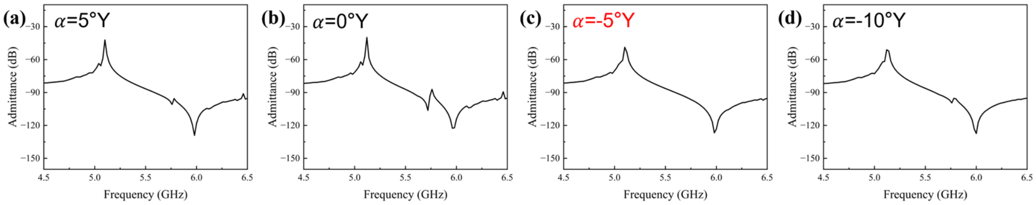

Figure S2. Simulated admittance responses of the BRR in different propagation directions: (a) +5°Y, (b) 0°Y, (c) -5°Y, and (d) -10°Y. The BRR also obtains the cleanest target Love-mode response along the -5°Y direction.

II. Fabrication Process of the ERR

The ERR fabrication process forms locally etched $LiNbO_3$ free edges while preserving the underlying SiC substrate. Figure S3 summarizes the process sequence, and the individual mask-transfer and electrode-fabrication steps are listed below.

1. A 200-nm-thick Cr layer was deposited by electron-beam evaporation and used as a hard-mask layer (Fig. S3a).
2. A 320-nm-thick SiO_2 layer was deposited by plasma-enhanced chemical vapor deposition (PECVD) and used as an intermediate mask layer (Fig. S3a).
3. Photolithography was performed on the SiO_2 layer to define the free-edge pattern (Fig. S3b).
4. The SiO_2 layer was etched by inductively coupled plasma reactive ion etching (ICP-RIE) to transfer the free-edge pattern into the SiO_2 layer (Fig. S3c).
5. After removal of the residual photoresist, the patterned SiO_2 layer was used as a mask, and the Cr layer was etched by ICP-RIE to further transfer the pattern into the Cr layer (Fig. S3d).
6. Using the patterned SiO_2/Cr stack as a composite hard mask, the 300-nm-thick $LiNbO_3$ film was etched by ICP-RIE using a CHF_3/Ar gas mixture until the underlying SiC substrate was exposed, thereby forming the free-edge structure (Fig. S3e).
7. The SiO_2 layer was removed using buffered oxide etchant (BOE) (Fig. S3f).
8. The Cr layer was removed by wet etching (Fig. S3g).
9. Finally, the IDT electrodes were patterned by electron-beam lithography (EBL), followed by metal deposition and lift-off (Fig. S3h).

The Cr/SiO_2 composite hard mask helps preserve the free-edge pattern during $LiNbO_3$ etching. Compared with direct etching through photoresist alone, the sequential transfer from photoresist to SiO_2, Cr, and $LiNbO_3$ reduces mask erosion and pattern

retreat during the final CHF_3/Ar ICP-RIE step. This process helps maintain the designed free-edge profile while exposing the SiC substrate only in the etched boundary region.

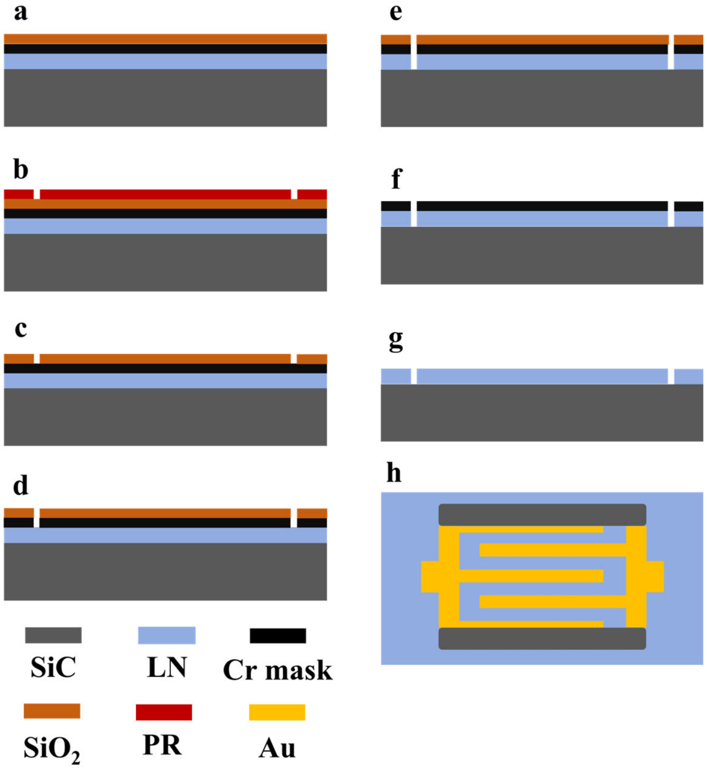

Figure S3. Schematic fabrication process of the ERR, including composite hard-mask formation, pattern transfer, $LiNbO_3$ etching, mask removal, and IDT electrode fabrication.

III. Influence of IDT Sidewall Angle on Resonator Performance

The IDT electrodes fabricated by electron-beam lithography, metal deposition, and lift-off may have nonideal sidewall profiles. To evaluate whether this electrode-sidewall geometry affects the resonator response, three models with IDT sidewall angles of 80°, 85°, and 90° were simulated. All other material parameters, geometric dimensions, and simulation settings were kept identical.

The simulated admittance responses show that the main Love-mode resonance remains nearly unchanged as the IDT sidewall angle varies from 80° to 90°. A slight shift in the antiresonance frequency is observed, which can be attributed to the small change in the effective electrode cross-section and metal loading. No obvious additional parasitic response is introduced within this sidewall-angle range. Therefore, the IDT sidewall angle is expected to have a weaker influence on the main resonator performance than the etched $LiNbO_3$ free-edge sidewall angle, which directly affects

acoustic reflection and lateral energy confinement.

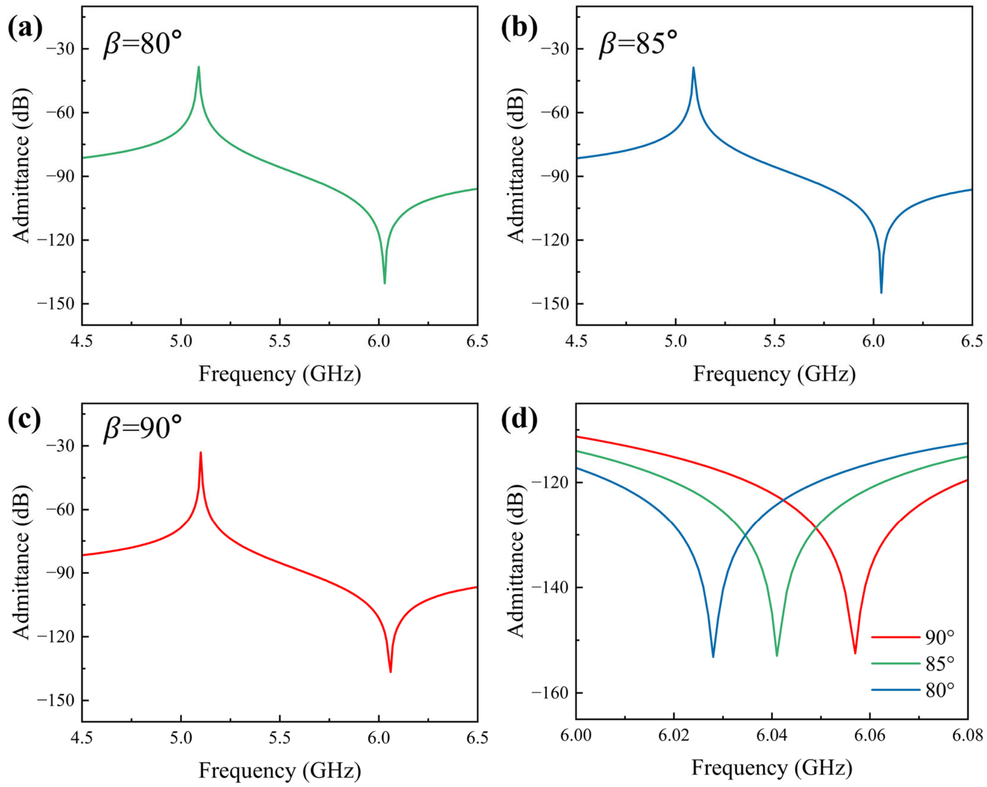

Figure S4. Simulated admittance responses of the resonator with different IDT sidewall angles. (a-c) Admittance responses for IDT sidewall angles of 80°, 85°, and 90°, respectively. (d) Enlarged view around the antiresonance frequency. The main resonance response remains nearly unchanged, while a slight shift in the antiresonance frequency is observed as the IDT sidewall angle varies.
